# Comparison of Students' Attitudes and Knowledge Regarding Functional Foods in Gastronomy, Food Science, and Nutrition Programs

**DOI:** 10.1002/fsn3.70321

**Published:** 2025-05-23

**Authors:** Şenay Burçin Alkan, Hilal Öz, Berna Madalı Kafes, Hasan Hüseyin Kara

**Affiliations:** ^1^ Department of Nutrition and Dietetics, Nezahat Keleşoğlu Faculty of Health Sciences Necmettin Erbakan University Konya Turkey; ^2^ Department of Gastronomy and Culinary Arts, School of Tourism and Hotel Management Mustafa Kemal University Hatay Turkey

**Keywords:** food engineering, functional food, gastronomy and culinary arts, nutrition and dietetics

## Abstract

The awareness and consumption of functional foods (FFs) have increased in recent years. To develop FFs and accurately inform consumers, fundamental knowledge of students enrolled in the Gastronomy and Culinary Arts (GCA), Food Engineering (FE), and Nutrition and Dietetics (ND) is essential. The aim of the study is to evaluate the attitudes and knowledge of students enrolled in the GCA, FE, and ND departments regarding FFs. The study was planned as a cross‐sectional study, and data were collected using an online survey form. Students were asked to evaluate various traditional and fortified foods in terms of their functional properties. Additionally, students' attitudes and knowledge toward FFs were assessed through questionnaires. The statistical analysis of the data was conducted using the Chi‐square test in SPSS version 22. The study included 348 students (34.8% from GCA, 33.0% from FE, 32.2% from ND departments). Among the participants, 36.5% enrolled in a course on FFs. The proportion of students who identified enriched foods as FFs was highest in the FE department, while students in the GCA department predominantly identified traditional foods as FFs. Students in the ND department commonly identified both traditional and enriched foods as FFs. Positive attitudes toward FFs were more prevalent among those who enrolled in the FFs course, and these students prioritized health claims, food composition, price, and safety when purchasing FFs. The study recommends incorporating FFs courses into the curricula to enhance students' knowledge and their ability to innovate and promote FFs in society.

## Graphical abstract

Students enrolled in the functional foods course, and those from the department of Nutrition and Dietetics demonstrated a high rate of identifying both traditional and enriched foods as functional foods. The attitudes of students who enrolled in the functional foods course toward functional foods were found to be more moderate.

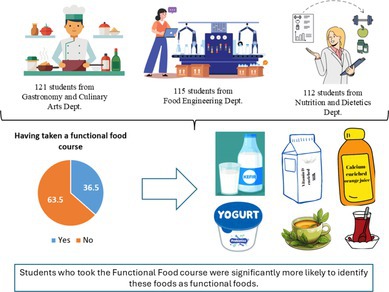

## 1 Introduction

The growing awareness of the relationship between diet and health has substantially influenced food choices in recent decades (Shah et al. 2025). As global concerns about chronic diseases continue to rise, individuals are increasingly prioritizing food products that provide health benefits beyond fundamental nutritional value (Shahnaz et al. 2024).Considering these trends, functional foods (FFs) have attracted significant scholarly and commercial interest due to their bioactive constituents, which are associated with health promotion and the prevention of disease (Dixit et al. 2022).

A common definition for FFs does not exist. The American Dietetic Association (ADA) defines FFs as items that can have positive effects on health when consumed regularly and in sufficient quantities as part of the diet (American Dietetic Association 1999). The ADA has divided FFs into four categories: traditional foods (such as garlic and tomatoes), modified foods (enriched foods), medical foods (formulas that do not contain phenylalanine), and foods for special dietary purposes (American Dietetic Association 2009). FFs are generally defined as “foods or nutritional components that, in addition to meeting the body's basic nutritional needs, provide additional physiological and metabolic benefits for humans, and are therefore effective in preventing diseases and promoting a healthier life.” (Topolska et al. 2021) The International Life Sciences Institute defines FFs as foods that have positive effects on health due to their physiologically active nutritional components (ILSI, n.d.).

Although the market and scientific interest in FFs continue to grow, knowledge and attitudes toward these products vary considerably across different educational backgrounds and fields of study (Arayici et al. 2020). Students enrolled in food‐ and nutrition‐related disciplines—such as Nutrition and Dietetics (ND), Food Science and Engineering (FE), and Gastronomy and Culinary Arts (GCA)—will play a crucial role in shaping consumer perceptions and promoting healthier dietary practices (Aktaç et al. 2022). Therefore, it is essential to conduct a comprehensive assessment of their knowledge and attitudes toward FFs.

In the literature, limited research has compared students' perspectives across different food‐related disciplines, particularly in Türkiye. Therefore, this study aims to reveal the attitudes, behaviors, and knowledge of university students studying GCA, FE, and ND in Türkiye regarding FFs. By conducting this study, it is aimed to provide individuals in higher education undergraduate programs (gastronomy, food, and nutrition) with information about FFs consumer trends and perceptions.

## 2 Methods

### 2.1 Participants

Undergraduate students from the GCA, FE, and ND departments at various universities across Türkiye participated in the research. An examination of the course curricula at the universities in Türkiye revealed that some departments did not include a course on FFs. In other departments, such a course was offered as either a compulsory or elective subject under titles such as “FFs” or “FFs and Health.”

The research data were collected using an online survey form. The survey was distributed through email and social networks. The study included students who were currently receiving formal education in GCA, FE, or ND at a university in Türkiye and who voluntarily participated in the survey. The study excluded students who were not studying in Türkiye, those enrolled in the open or distance education system, graduate students, and students from other departments.

The sample size was determined using the G*Power 3.1.9.2 software (Faul et al. 2007). Assuming an equal distribution of students who enrolled in the FFs course or not, it was calculated that each group would consist of a minimum of 105 participants, based on an effect size of *d* = 0.5, a significance level of *α* = 0.05, and a statistical power of 0.95.

### 2.2 Survey Form

Participants were surveyed on various aspects, including general demographics (department, class, gender, age); physician‐diagnosed diseases; dietary habits; usage of dietary supplements; and enrollment in FFs courses. They were also required to assess kefir, strawberries, broccoli, vitamin D‐fortified milk, calcium‐fortified orange juice, oats, probiotic yogurt, green tea, and black tea concerning their functional nutrient content. These nine foods were selected by researchers based on the FF definition and examples in two practical guides of the Academy of Nutrition and Dietetics (American Dietetic Association 1999, 2009). The evaluation of students' perspectives on FFs utilized the expressions from Annunziata and Vecchio's study (Annunziata and Vecchio 2011). Additionally, students were questioned about the attributes they prioritize when purchasing FFs.

### 2.3 Statistical Analysis

The statistical analysis of the data was conducted using SPSS version 22. Categorical data are presented as frequencies and percentages. The Chi‐square test was used to evaluate students' assessments of food in terms of FF, their opinions and attitudes toward FFs, and the attributes they consider when purchasing FFs. Results with *p*‐values of less than 0.05 were deemed statistically significant.

### 2.4 Ethics Statement

Ethical approval for this study was obtained from Toros University Scientific Research and Publication Ethics Committee (2022/19, 28 Jan 2022). Informed consent was obtained from all individual participants included in the study.

## 3 Results

A total of 348 students participated in the study. Of these, 34.8% were enrolled in the GCA department, 33.0% in the FE department, and 32.2% in the ND department. The majority of the students were studying at Necmettin Erbakan University, Selçuk University, Mustafa Kemal University, and Adnan Menderes University. Additionally, 36.5% of the participants enrollment in FFs course. The mean age of the students was 22.3 ± 3.7 years (Table 1).

**TABLE 1:** General characteristics of students.

	*n*	%
Department		
Gastronomy and Culinary Arts (GCA)	121	34.8
Food Engineering (FE)	115	33.0
Nutrition and Dietetics (ND)	112	32.2
Universities		
Necmettin Erbakan University, Konya	131	37.6
Selçuk University, Konya	49	14.1
Mustafa Kemal University, Hatay	25	7.2
Adnan Menderes University, Aydın	25	7.2
Yüzüncü Yıl University, Van	18	5.2
Çukurova University, Adana	18	5.2
Giresun University	12	3.4
Gaziantep University	11	3.2
Others	59	16.9
Gender		
Female	297	85.3
Male	51	14.7
Grade		
Freshman	72	20.7
Sophomore	115	33.0
Junior	56	16.1
Senior	105	30.2
Enrollment in the functional foods course		
Yes	127	36.5
No	221	63.5
Age (year, $\bar{X}$ ± SD)	22.3 ± 3.7	

Among the students, 37.1% reported having a diagnosed disease. The three most prevalent conditions are ophthalmic diseases (34.9%), vitamin D deficiency (28.7%), and allergies (26.6%). Additionally, 9.2% of the students indicated that they were following a diet, with the most common type was a weight‐loss diet (59.4%). Furthermore, 31.0% of the students reported using dietary supplements, with the most frequently consumed supplements being vitamin D (57.4%), vitamin B_12_ (56.5%), and iron (31.5%) (Table 2).

**TABLE 2:** Health, dietary habits, and dietary supplement usage status of students.

	*n*	%
Diagnosis of disease		
Yes	129	37.1
No	219	62.9
Diseases		
Ophthalmic diseases	45	34.9
Vitamin D deficiency	37	28.7
Allergy	33	26.6
Anemia (iron)	31	24.0
Anemia (vitamin B_12_ deficiency)	25	19.4
Polycystic ovary syndrome	6	4.7
Depression	5	3.9
Ulcerative colitis	4	3.1
Diabetes mellitus	3	2.3
Cancer	2	1.6
Epilepsy	1	0.8
Celiac disease	1	0.8
Multiple sclerosis	1	0.8
Hypertension	1	0.8
Dieting		
Yes	32	9.2
No	316	90.8
Types of diet		
Weight loss diet	19	59.4
Gluten‐free diet	5	15.6
Low fat low cholesterol diet	4	12.5
High protein diet	4	12.5
Usage of dietary supplements		
Yes	108	31.0
No	240	69.0
Dietary supplements^a^		
Vitamin D	62	57.4
Vitamin B_12_	61	56.5
Iron	34	31.5
Fish oil	17	15.7
Zinc	14	12.9
Magnesium	12	11.1
Multivitamin	8	7.4
Multimineral	3	2.8
Calcium	3	2.8
Protein powder	3	2.8
Collagen	2	1.9

^a^ Some students have used multiple nutritional supplements.

Students were asked to evaluate whether the foods were FFs or not. The percentage of ND (39.3% and 42.0%) and GCA students (33.9% and 44.6%) who consider strawberries and broccoli as FFs was significantly higher than the rate of FE students (23.5% and 25.2%) (*p* < 0.05). The proportion of students from the departments of ND (74.1%, 69.6%, and 75.0%) and FE (74.8%, 75.7%, and 83.3%) who assessed vitamin D‐enriched milk, calcium‐enriched orange juice, and probiotic yogurt as FFs was significantly higher than that of students from the department of GCA (46.3%, 49.6%, and 52.9%) (*p* < 0.05). The percentage of students from the department of ND who evaluated oats (41.1%) as a FF was significantly higher than that of students from the department of FE (23.5%) (*p* < 0.05).

The proportion of ND students (38.4%) who assessed green tea as a FF was significantly greater than that of FE (26.1%) and GCA students (24.0%) (*p* < 0.05). The percentage of sophomore students (40.9%) who evaluated strawberries as a FF was significantly higher than that of senior students (22.9%) (*p* < 0.05). The proportions of freshman, sophomore and junior students (43.1%, 47.0%, and 41.1%, respectively) who evaluated broccoli as a FF were significantly higher than that of senior students (21.0%) (*p* < 0.05). The percentages of sophomore, junior, and senior students (67.0% and 63.5%; 69.6% and 73.2%; 72.4% and 76.2%, respectively) who evaluated milk enriched with vitamin D and orange juice enriched with calcium as FFs were significantly higher than those of freshman students (45.8% and 43.1%) (*p* < 0.05). The proportions of freshman and sophomore students (44.4% and 39.1%, respectively) who evaluated oats as a FF were significantly higher than that of senior students (21.9%) (*p* < 0.05). The percentage of senior students (85.6%) who evaluated probiotic yogurt as a FF was significantly higher than that of freshman and sophomore students (44.4% and 68.7%, respectively). The proportion of sophomore students was significantly higher than that of freshman students (*p* < 0.05). Among the students who enrolled in the FFs course, the percentage who considered kefir (69.3%), vitamin D‐enriched milk (77.2%), calcium‐enriched orange juice (79.5%), probiotic yogurt (84.1%), green tea (43.3%), and black tea (27.6%) as FFs was significantly higher than those of students who did not enroll the course (kefir: 54.3%, vitamin D‐enriched milk: 57.5%, calcium‐enriched orange juice: 56.1%, probiotic yogurt: 62.0%, green tea: 21.3%, and black tea: 16.3%; *p* < 0.05). Among the students who were diagnosed with the disease by a physician, the proportion of those who evaluated vitamin D‐enriched milk (71.3%) as a FF was significantly higher (*p* < 0.05) than that of undiagnosed students (60.7%). No significant differences were found between the rates of evaluating foods in terms of FFs according to gender and dietary supplement usage (Table 3).

**TABLE 3:** The assessment of food as functional foods conducted by students.

	Kefir		Strawberry		Broccoli		Vitamin D enriched milk		Calcium‐enriched orange juice	
	Functional food	Not a functional food	Functional food	Not a functional food	Functional food	Not a functional food	Functional food	Not a functional food	Functional food	Not a functional food
Departments										
GCA	70 (57.9%)	51 (42.1%)	41 (33.9%)	80 (66.1%)	54 (44.6%)	67 (55.4%)	56 (46.3%)	65 (53.7%)	60 (49.6%)	61 (50.4%)
FE	69 (60.0%)	46 (40.0%)	27 (23.5%)	88 (76.5%)	29 (25.2%)	86 (74.8%)	86 (74.8%)	29 (25.2%)	87 (75.7%)	28 (24.3%)
ND	67 (59.8%)	45 (40.2%)	44 (39.3%)	68 (60.7%)	47 (42.0%)	65 (58.0%)	83 (74.1%)	29 (25.9%)	78 (69.6%)	34 (30.4%)
	*χ* ^2^ = 0.140, *p* = 0.933		***χ*** ^**2**^ **= 6.742, *p* = 0.034**		***χ*** ^**2**^ **= 10.992, *p* = 0.004**		***χ*** ^**2**^ **= 27.416, *p* < 0.001**		***χ*** ^**2**^ **= 19.327, *p* < 0.001**	
Gender										
Female	175 (58.9%)	122 (41.1%)	101 (34.0%)	196 (66.0%)	115 (38.7%)	182 (61.3%)	197 (66.3%)	100 (33.7%)	195 (65.7%)	102 (34.3%)
Male	31 (60.8%)	20 (39.2%)	11 (21.6%)	40 (78.4%)	15 (29.4%)	36 (70.6%)	28 (54.9%)	23 (45.1%)	30 (58.8%)	21 (41.2%)
	*χ* ^2^ = 0.062, *p* = 0.803		*χ* ^2^ = 3.085, *p* = 0.079		*χ* ^2^ = 1.612, *p* = 0.204		*χ* ^2^ = 2.487, *p* = 0.115		*χ* ^2^ = 0.889, *p* = 0.346	
Grade										
Freshman	35 (48.6%)	37 (51.4%)	22 (30.6%)	50 (69.4%)	31 (43.1%)	41 (56.9%)	33 (45.8%)	39 (54.2%)	31 (43.1%)	41 (56.9%)
Sophomore	69 (60.0%)	46 (40.0%)	47 (40.9%)	68 (59.1%)	54 (47.0%)	61 (53.0%)	77 (67.0%)	38 (33.0%)	73 (63.5%)	42 (36.5%)
Junior	38 (67.9%)	18 (32.1%)	19 (33.9%)	37 (66.1%)	23 (41.1%)	33 (58.9%)	39 (69.6%)	17 (30.4%)	41 (73.2%)	15 (26.8%)
Senior	64 (61.0%)	41 (39.0%)	24 (22.9%)	81 (77.1%)	22 (21.0%)	83 (79.0%)	76 (72.4%)	29 (27.6%)	80 (76.2%)	25 (23.8%)
	*χ* ^2^ = 5.244, *p* = 0.155		***χ*** ^**2**^ **= 8.325, *p* = 0.040**		***χ*** ^**2**^ **= 17.933, *p* < 0.001**		*χ* ^**2**^ **= 14.780, *p* = 0.002**		***χ*** ^**2**^ **= 22.678, *p* < 0.001**	
Taking the functional foods course										
Yes	88 (69.3%)	39 (30.7%)	45 (34.4%)	82 (64.6%)	50 (39.4%)	77 (60.6%)	98 (77.2%)	29 (22.8%)	101 (79.5%)	26 (20.5%)
No	118 (54.3%)	103 (46.6%)	67 (30.3%)	154 (69.7%)	80 (36.2%)	141 (63.8%)	127 (57.5%)	94 (42.5%)	124 (56.1%)	97 (43.9%)
	***χ*** ^**2**^ **= 8.439, *p* = 0.004**		*X* ^2^ = 0.967, *p* = 0.325		*X* ^2^ = 0.347, *p* = 0.556		***χ*** ^**2**^ **= 13.693, *p* < 0.001**		***χ*** ^**2**^ **= 19.356, *p* < 0.001**	
Diagnosis of disease										
Yes	69 (53.5%)	60 (46.5%)	40 (31.0%)	89 (69.0%)	47 (36.4%)	82 (63.6%)	92 (71.3%)	37 (28.7%)	87 (67.4%)	42 (32.6%)
No	137 (62.6%)	82 (37.4%)	72 (32.9%)	147 (67.1)	83 (37.9%)	136 (62.1%)	133 (60.7%)	86 (39.3%)	138 (63.0%)	81 (37.0%)
	*X* ^2^ = 2.764, *p* = 0.096		*X* ^2^ = 0.130, *p* = 0.719		*X* ^2^ = 0.074, *p* = 0.785		***X*** ^**2**^ **= 3.982, *p* = 0.046**		*X* ^2^ = 0.697, *p* = 0.404	
Dietary supplement										
Yes	62 (57.4%)	46 (42.6%)	39 (36.1%)	69 (63.9%)	41 (38.0%)	67 (62.0%)	77 (71.3%)	31 (28.7%)	75 (69.4%)	33 (30.6%)
No	144 (60.0%)	96 (40.0%)	73 (30.4%)	167 (69.6%)	89 (37.1%)	151 (62.9%)	148 (61.7%)	92 (38.3%)	150 (62.5%)	90 (37.5%)
	*X* ^2^ = 0.207, *p* = 0.649		*X* ^2^ = 1.107, *p* = 0.293		*X* ^2^ = 0.025, *p* = 0.875		*X* ^2^ = 3.022, *p* = 0.082		*X* ^2^ = 1.572, *p* = 0.120	

	Oat		Probiotic yogurt		Green tea		Black tea	
	Functional food	Not a functional food	Functional food	Not a functional food	Functional food	Not a functional food	Functional food	Not a functional food
Departments								
GCA	44 (36.4%)	77 (63.6%)	64 (52.9%)	57 (47.1%)	29 (24.0%)	92 (76.0%)	23 (19.0%)	98 (81.0%)
FE	27 (23.5%)	88 (76.5%)	95 (83.3%)	19 (16.7%)	30 (26.1%)	85 (73.9%)	20 (17.4%)	95 (82.6%)
ND	46 (41.1%)	66 (58.9%)	84 (75.0%)	28 (25.0%)	43 (38.4%)	69 (61.6%)	28 (25.0%)	84 (75.0%)
	***X*** ^**2**^ **= 8.495, *p* = 0.014**		***X*** ^**2**^ **= 27.862, *p* < 0.001**		***X*** ^**2**^ **= 6.703, *p* = 0.035**		*X* ^2^ = 2.245, *p* = 0.326	
Gender								
Female	102 (34.3%)	195 (65.7%)	212 (71.6%)	84 (28.4%)	86 (29.0%)	211 (71.0%)	60 (20.2%)	237 (79.8%)
Male	15 (29.4%)	36 (70.6%)	31 (60.8%)	20 (39.2%)	16 (31.4%)	35 (68.6%)	11 (21.6%)	40 (78.4%)
	*X* ^2^ = 0.474, *p* = 0.491		*X* ^2^ = 2.434, *p* = 0.119		*X* ^2^ = 0.123, *p* = 0.726		*X* ^2^ = 0.050, *p* = 0.823	
Grade								
Freshman	32 (44.4%)	40 (55.6%)	32 (44.4%)	40 (55.6%)	18 (25.0%)	54 (75.0%)	15 (20.8%)	57 (79.2%)
Sophomore	45 (39.1%)	70 (60.9%)	79 (68.7%)	39 (31.3%)	35 (30.4%)	80 (69.6%)	28 (24.3%)	87 (75.7%)
Junior	17 (30.4%)	39 (69.6%)	43 (76.8%)	13 (23.2%)	23 (41.1%)	33 (58.9%)	15 (26.8%)	41 (73.2%)
Senior	23 (21.9%)	82 (78.1%)	89 (85.6%)	15 (14.4%)	26 (24.8%)	79 (75.2%)	13 (12.4%)	92 (87.6%)
	***X*** ^**2**^ **= 12.069, *p* = 0.007**		***X*** ^**2**^ **= 35.749, *p* < 0.001**		*X* ^2^ = 5.503, *p* = 0.1380		*X* ^2^ = 6.676, *p* = 0.083	
Enrollment in the functional foods course								
Yes	46 (36.2%)	81 (63.8%)	106 (84.1%)	20 (15.9%)	55 (43.3%)	72 (56.7%)	35 (27.6%)	92 (72.4%)
No	71 (32.1%)	150 (67.9%)	137 (62.0%)	84 (38.0%)	47 (21.3%)	174 (78.7%)	36 (16.3%)	185 (83.7%)
	*X* ^2^ = 0.606, *p* = 0.436		***X*** ^**2**^ **= 18.735, *p* < 0.001**		***X*** ^**2**^ **= 18.909, *p* < 0.001**		***X*** ^**2**^ **= 6.307, *p* = 0.012**	
Diagnosis of disease								
Yes	39 (30.2%)	90 (69.8%)	92 (71.3%)	37 (28.7%)	34 (26.4%)	95 (73.6%)	24 (18.6%)	105 (81.4%)
No	78 (35.6%)	141 (64.4%)	151 (69.3%)	67 (0.7%)	68 (31.1%)	151 (68.9%)	47 (61.5%)	172 (78.5%)
	*X* ^2^ = 1.054, *p* = 0.304		*X* ^2^ = 0.163, *p* = 0.687		*X* ^2^ = 0.863, *p* = 0.353		*X* ^2^ = 0.408, *p* = 0.523	
Dietary supplement								
Yes	37 (34.3%)	71 (65.7%)	78 (72.9%)	29 (27.1%)	32 (29.6%)	76 (70.4%)	22 (20.4%)	86 (79.6%)
No	80 (33.3%)	160 (66.7%)	165 (68.8%)	75 (31.3%)	70 (29.2%)	170 (70.8%)	49 (20.4%)	191 (79.6%)
	*X* ^2^ = 0.029, *p* = 0.866		*X* ^2^ = 0.606, *p* = 0.436		*X* ^2^ = 0.008, *p* = 0.930		*X* ^2^ = 0.000 *p* = 0.992	

*Note:* The bolded values indicate statistically significant differences between groups.

The opinions and thoughts of the students about FFs were evaluated according to their status of enrollment in the FFs course. Among the students who enrolled in the FFs course, the statements “FFs are likely to have a beneficial impact on my personal health” (57.5%), “FFs promote my well‐being” (55.1%), “FFs make it easier to follow a healthy lifestyle” (43.3%), “I enjoy eating FFs”. (32.3%), “FFs are top science‐based products” (32.3%), and “I trust the information given about health effects” (25.2%) were significantly higher compared to the students who did not enroll in the course (39.8%, 40.7%, 26.7%, 18.6%, 15.8%, and 14.0%, respectively). The proportion of students who did not enroll in the course and responded, “I am undecided” to the statement “The information on the label is difficult to understand” (38.5%) was significantly higher than that of students who enrolled in the course (26.8%) (*p* < 0.05).

Among the students enrolled in the FFs course, the proportions of those who strongly disagree with the statements “FFs are intended only for those who have health problems” (72.4%), “FFs taste worse than conventional foods” (34.6%), “The information on the label is difficult to understand” (31.5%), “FFs are simply a passing fad” (52.0%), “FFs are completely unnecessary” (67.7%), and “For a healthy person it is pointless to use FFs” (58.3%) were significantly higher (*p* < 0.05) than those of students who did not enroll in the course (52.5%, 21.7%, 17.2%, 34.8%, 42.1%, and 33.5%, respectively). Among the students who enrolled in the FFs course, the proportion of those who purchased FFs (94.5%) was significantly higher than among those who did not enroll in the course (77.8%). Additionally, among the students who enrolled in the course, the proportions of those who paid attention to health claims (73.3%), food composition (86.7%), price (72.5%), and safety (73.3%) when purchasing FFs were significantly higher than those among students who did not enroll in the course (61.0%, 75.6%, 58.7%, and 55.2%, respectively) (Table 5).

## 4 Discussion

Interest in nutrition, a modifiable risk factor, has increased to improve quality of life and protect health in public. The concept of FFs, recognized as an alternative and shown by studies to have health‐improving effects, has become particularly important for individuals working in fields related to food and nutrition. Consequently, this study was conducted to determine the understanding of the FFs concept, purchasing preferences, and knowledge of students in university programs related to food and nutrition, excluding health professionals.

The study found that a high proportion of students in the department of FE identified enriched foods (vitamin D‐enriched milk, calcium‐enriched orange juice, and probiotic yogurt) as FFs. Conversely, students in the department of GCA predominantly defined traditional foods as FFs. Among students in the department of ND, the proportion of those who defined both traditional foods (strawberries, broccoli, oats, and green tea) and enriched foods as FFs was notably high (Table 3). This suggests that students in the department of ND have greater knowledge of the subject compared to those in other departments. This disparity is likely attributable to the curriculum of the department of ND, which places a greater emphasis on food and health‐related subjects. In the study conducted by Arayici et al. (2020), it was found that students in the department of ND demonstrated a higher level of knowledge about FFs. The lower level of knowledge about FFs in the other departments included in the study might be attributed to the prioritization of concerns regarding subjective quality and market availability, driven by commercial interests (Aygül et al. 2018). Studies in the literature have shown that the knowledge level of individuals working in the health field about FFs is higher compared to those in other departments (Korzen‐Bohr and Jensen 2006; Landström et al. 2009). In a study conducted by Aygül et al. (2018), it was shown that participants were more likely to trust information about FFs when it was provided by dietitians rather than by individuals working in the food industry. Similarly, in a study by Morawska et al. (2016), it was shown that students in the department of ND have higher knowledge levels about FFs compared to students in the Faculty of Pharmacy. This difference was attributed to the department of ND offering more courses related to both health and food and nutrition.

The study found that students' knowledge of FFs increased in more advanced grades (Table 3). Despite varying results, it has been reported that as the level of grade increases, the number of individuals' knowledge about FFs also increases (Demir 2018; Derya 2015).

Similarly, in a study conducted on ND students in Türkiye, it was reported that the mean knowledge score about FFs was significantly lower in freshman students (23.7 ± 5.0) compared to senior students (29.4 ± 3.1) (Demir and Aktaş 2018). The results of the present study also support this finding. Additionally, in a study by Annunziata and Vecchio (2011), it was reported that the frequency of consuming FFs increases as the level of education rises (Annunziata and Vecchio 2011). In the study, it was found that gender and the use of dietary supplements did not affect the knowledge about FFs (Table 3). Similarly, Annunziata and Vecchio (2011) reported that gender did not influence the knowledge about FFs, but that knowledge increased in the presence of a health‐related condition (Annunziata and Vecchio 2011). It is hypothesized that the findings related to dietary supplements are influenced by the lack of inquiries regarding participants' use of these supplements for disease prevention or health enhancement.

There are a limited number of studies in the literature evaluating the effect of taking courses on FFs on attitudes toward these foods. According to the results of this research, it could be said that the attitudes of students who have enrolled in the FFs course are more positive toward FFs compared to those who have not enroll in the course (Table 4). In a study conducted among university students, Schnettler et al. (2016) stated that insufficient knowledge about FFs negatively affects both the belief in their effects and their consumption (Schnettler et al. 2016).

**TABLE 4:** Assessment of students' attitudes toward functional foods.

	Students enrolled in the functional foods course (*n*: 127)					Students who did not enroll in the functional foods course (*n*: 221)					*p*
	Strongly disagree	Disagree	Undecided	Agree	Strongly agree	Strongly disagree	Disagree	Undecided	Agree	Strongly agree	
FFs are likely to have a beneficial impact on my personal health	5 (3.9%)	1 (0.8%)	12 (9.4%)	36 (28.3%)	73 (57.5%)	12 (5.4%)	13 (5.9%)	43 (19.5%)	65 (29.4%)	88 (39.8%)	***X*** ^**2**^ **= 16.153** ***p* = 0.003**
FFs can repair the damage caused by an unhealthy diet	7 (5.5%)	18 (14.2%)	31 (24.4%)	38 (29.9%)	33 (26.0%)	16 (7.2%)	17 (7.7%)	67 (30.3%)	82 (37.1%)	39 (17.6%)	*X* ^2^ = 8.6448 *p* = 0.071
FFs are intended only for those who have health problems	92 (72.4%)	20 (15.7%)	8 (6.3%)	2 (1.6%)	5 (3.9%)	116 (52.5%)	66 (29.9%)	26 (11.8%)	5 (2.3%)	8 (3.6%)	***X*** ^**2**^ **= 14.552** ***p* = 0.006**
FFs promote my well‐being	6 (4.7%)	1 (0.8%)	10 (7.9%)	40 (31.5%)	70 (55.1%)	15 (6.8%)	8 (3.6%)	35 (15.8%)	73 (33.0%)	90 (40.7%)	***X*** ^**2**^ **= 10.719** ***p* = 0.030**
I can prevent disease by eating FFs regularly	7 (5.5%)	9 (7.1%)	39 (30.7%)	44 (34.6%)	28 (22.0%)	11 (5.0%)	30 (13.6%)	67 (30.3%)	82 (37.1%)	31 (14.0%)	*X* ^2^ = 6.273 *p* = 0.180
Functional foods make it easier to follow a healthy lifestyle	3 (2.5%)	1 (0.8%)	17 (13.4%)	51 (40.2%)	55 (43.3%)	12 (5.4%)	20 (9.0%)	46 (20.8%)	84 (38.0%)	59 (26.7%)	***X*** ^**2**^ **= 20.232** ***p* < 0.001**
FFs contain unnatural substances	48 (37.8%)	41 (32.3%)	24 (18.9%)	8 (6.3%)	6 (4.7%)	62 (28.1%)	72 (32.8%)	62 (28.1%)	17 (7.7%)	8 (3.6%)	*X* ^2^ = 5.622 *p* = 0.229
FFs are more expensive than conventional	12 (9.4%)	13 (10.2%)	45 (35.4%)	39 (30.7%)	18 (14.2%)	21 (9.5%)	36 (16.3%)	83 (37.6%)	50 (22.6%)	31 (14.0%)	*X* ^2^ = 4.260 *p* = 0.372
It is not easy to find these products	30 (23.6%)	42 (33.1%)	30 (23.6%)	18 (14.2%)	7 (5.5%)	35 (15.8%)	70 (31.7%)	75 (33.9%)	26 (11.8%)	15 (6.8%)	*X* ^2^ = 6.087 *p* = 0.193
The range of FFs on the market is limited	22 (17.3%)	21 (16.5%)	44 (34.6%)	25 (19.7%)	15 (11.8%)	25 (11.3%)	59 (26.7%)	83 (37.6%)	34 (15.4%)	20 (9.0%)	*X* ^2^ = 7.458 *p* = 0.114
It's difficult to distinguish functional from conventional foods	31 (24.4%)	41 (32.3%)	38 (29.9%)	12 (9.4%)	5 (3.9%)	36 (16.3%)	88 (39.8%)	68 (30.8%)	19 (8.6%)	10 (4.5%)	*X* ^2^ = 4.147 *p* = 0.387
I enjoy eating FFs	4 (3.1%)	7 (5.5%)	33 (26.0%)	42 (33.1%)	41 (32.3%)	18 (8.1%)	29 (13.1%)	63 (28.5%)	70 (31.7%)	41 (18.6%)	***X*** ^**2**^ **= 14.387** ***p* = 0.006**
FFs taste worse than conventional foods	44 (34.6%)	39 (30.7%)	30 (23.6%)	11 (8.7%)	3 (2.4%)	48 (21.7%)	76 (34.4%)	72 (32.6%)	19 (8.6%)	6 (2.7%)	*X* ^2^ = 7.675 *p* = 0.104
The information on the label is difficult to understand	40 (31.5%)	40 (31.5%)	34 (26.8%)	8 (6.3%)	5 (3.9%)	38 (17.2%)	69 (31.2%)	85 (38.5%)	19 (8.6%)	10 (4.5%)	***X*** ^**2**^ **= 11.198** ***p* = 0.024**
FFs are simply a passing fad	66 (52.0%)	34 (26.8%)	18 (14.2%)	3 (2.4%)	6 (4.7%)	77 (34.8%)	80 (36.2%)	48 (21.7%)	7 (3.2%)	9 (4.1%)	***X*** ^**2**^ **= 10.629** ***p* = 0.031**
FFs are completely unnecessary	86 (67.7%)	23 (18.1%)	8 (6.2%)	3 (2.4%)	7 (5.5%)	93 (42.1%)	80 (36.2%)	34 (15.4%)	7 (3.2%)	7 (3.2%)	***X*** ^**2**^ **= 26.020** ***p* < 0.001**
For a healthy person it is pointless to use FFs	77 (60.6%)	30 (23.6%)	12 (9.4%)	4 (3.1%)	4 (3.1%)	92 (41.6%)	72 (32.6%)	42 (19.0%)	11 (5.0%)	4 (1.8%)	***X*** ^**2**^ **= 14.204** ***p* = 0.007**
FFs are top science‐based products	6 (4.7%)	9 (7.1%)	33 (26.0%)	38 (29.9%)	41 (32.3%)	17 (7.7%)	31 (14.0%)	62 (28.1%)	76 (34.4%)	35 (15.8%)	***X*** ^**2**^ **= 15.062** ***p* = 0.005**

	Students taking functional foods course					Students who did not take functional foods course					*p*
	Strongly disagree	Disagree	Undecided	Agree	Strongly agree	Strongly disagree	Disagree	Undecided	Agree	Strongly agree	
I fear that FFs may have side effects	30 (23.6%)	33 (26.0%)	44 (34.6%)	16 (12.6%)	4 (3.1%)	38 (17.2%)	70 (31.7%)	81 (36.7%)	24 (10.9%)	8 (3.6%)	*X* ^2^ = 2.942 *p* = 0.568
I am cautious about the consumption of FFs	23 (18.1%)	38 (29.9%)	37 (29.1%)	20 (15.7%)	9 (7.1%)	32 (14.5%)	51 (23.1%)	81 (36.7%)	45 (20.4%)	12 (5.4%)	*X* ^2^ = 4.780 *p* = 0.311
I do not believe FF properties	74 (58.3%)	33 (26.0%)	12 (9.4%)	3 (2.4%)	5 (3.9%)	74 (33.5%)	88 (39.8%)	44 (19.9%)	12 (5.4%)	3 (1.4%)	***X*** ^**2**^ **= 25.668** ***p* < 0.001**
FFs are completely safe	13 (10.2%)	28 (22.0%)	50 (39.4%)	28 (22.0%)	8 (6.3%)	18 (8.1%)	55 (24.9%)	95 (43.0%)	43 (19.5%)	10 (4.5%)	*X* ^2^ = 1.678 *p* = 0.795
If used in excess, FFs can be harmful to health	6 (4.7%)	10 (7.9%)	24 (18.9%)	45 (35.4%)	42 (33.1%)	16 (7.2%)	32 (14.5%)	45 (20.4%)	67 (30.3%)	61 (27.6%)	*X* ^2^ = 5.281 *p* = 0.260
I trust the information given about health effects	5 (3.9%)	7 (5.5%)	39 (30.7%)	44 (34.6%)	32 (25.2%)	11 (5.0%)	31 (14.0%)	78 (35.3%)	70 (31.7%)	31 (14.0%)	***X*** ^**2**^ **= 11.826** ***p* = 0.019**

Abbreviation: FFs, functional foods.

Considering that nutrition has positive effects on health, it is stated that consumers particularly prefer FFs due to their health‐promoting benefits (Conroy et al. 2021; Nong et al. 2022). This finding is supported by a study conducted by Kraus et al. (2015), which reported that consumers prefer to purchase FFs because they believe these foods have positive effects on health and enhance quality of life (Kraus 2015).

Awareness of the health effects of FFs is a significant factor influencing their purchase (Baker et al. 2022). In this study, the rate of purchasing FFs was found to be significantly higher among the students who enrolled in the FFs course. However, it was determined that they paid more attention to health claims, food composition, price, and safety (Table 5).

**TABLE 5:** Key factors considered by students when purchasing functional foods.

	Students enrolled in the functional foods course (*n*: 127)	Students who did not enroll in the functional foods course (*n*: 221)	*p*
Purchasing			
Yes	120 (94.5%)	172 (77.8%)	***X*** ^**2**^ **= 16.579** ***p* < 0.001**
No	7 (5.5%)	49 (22.2%)	
Label			
Yes	113 (94.2%)	154 (89.5%)	*X* ^2^ = 1.937 *p* = 0.164
No	7 (5.89)	18 (10.5%)	
Health claim			
Yes	88 (73.3%)	105 (61.0%)	***X*** ^**2**^ **= 4.762** ***p* = 0.029**
No	32 (26.7%)	67 (39.0%)	
Nutrition claim			
Yes	68 (56.7%)	83 (48.3%)	*X* ^2^ = 2.003 *p* = 0.157
No	52 (43.3%)	89 (51.7%)	
Food composition			
Yes	104 (86.7%)	130 (75.6%)	***X*** ^**2**^ **= 5.457** ***p* = 0.019**
No	16 (13.3%)	42 (24.4%)	
Price			
Yes	87 (72.5%)	101 (58.7%)	***X*** ^**2**^ **= 5.852** ***p* = 0.016**
No	33 (27.5%)	71 (41.3%)	
Safety			
Yes	88 (73.3%)	95 (55.2%)	***X*** ^**2**^ **= 9.899** ***p* = 0.002**
No	32 (26.7%)	77 (44.8%)	

*Note:* The bolded values indicate statistically significant differences between groups.

The study demonstrated that label information and health claim influence purchasing behavior (Table 5). Similarly, previous studies indicate that health‐related claims enhance the likelihood of purchasing FFs (Masson et al. 2016; Vecchio et al. 2016). When the studies on the issue are examined, it is reported that they tend to buy FFs with the idea that they have protective effects especially against cardiovascular diseases and cancer (Ares et al. 2008; van Kleef et al. 2005).

## 5 Implications for Research and Practice

Students enrolled in the FFs course, and those from the department of ND demonstrated a high rate of identifying both traditional and enriched foods as FFs. The attitudes of students who enrolled in the FFs course toward FFs were found to be more moderate. Additionally, these students paid greater attention to health claims, food composition, price, and safety when purchasing FFs.

The departments of GCA, FE, and ND have crucial roles in advancing public health. These departments have significant potential to collaborate in enhancing public knowledge about FFs, developing new FFs, and presenting them to consumers. To ensure that students in these departments acquire adequate knowledge, it is recommended to incorporate a course on FFs into their curriculum and to encourage students to enroll in this course.

## Author Contributions

**Şenay Burçin Alkan:** conceptualization (equal), formal analysis (equal), investigation (equal), methodology (equal), resources (equal), software (equal), writing – original draft (equal), writing – review and editing (equal). **Hilal Öz:** conceptualization (equal), data curation (equal), investigation (equal), resources (equal), software (equal), writing – original draft (equal). **Berna Madalı Kafes:** conceptualization (equal), formal analysis (equal), investigation (equal), methodology (equal), writing – review and editing (equal). **Hasan Hüseyin Kara:** conceptualization (equal), investigation (equal), methodology (equal), supervision (equal), writing – review and editing (equal).

## Disclosure

The authors have nothing to report.

## Ethics Statement

Ethical approval for this study was obtained from Toros University Scientific Research and Publication Ethics Committee (2022/19, January 28, 2022). Informed consent was obtained from all individual participants included in the study.

## Conflicts of Interest

The authors declare no conflicts of interest.

## Data Availability

The data that support the findings of this study are available from the corresponding author upon reasonable request.
